# Online sex-seeking among Chinese heterosexual men who seek care in sexually transmitted infections clinics: a cross-sectional study

**DOI:** 10.1186/s13690-022-00903-5

**Published:** 2022-06-01

**Authors:** Changchang Li, Weiming Tang, Hung Chak Ho, Peizhen Zhao, Lei Chen, Yajie Wang, Mingzhou Xiong, Bin Yang, Heping Zheng, Cheng Wang

**Affiliations:** 1 Department of Sexually Transmitted Disease Prevention and Control, Dermatology Hospital of Southern Medical University, Institute for Global Health and Sexually Transmitted Infections, Southern Medical University, Guangzhou, 510095 China; 2 Institute for Global Health and Sexually Transmitted Infections, Southern Medical Universi-Ty, Guangzhou, China; 3 The University of Hong Kong, Hong Kong, China

**Keywords:** Online sex-seeking, Sexual behavior, Heterosexual men, Sexually transmitted infection

## Abstract

**Background:**

The Internet has become an important virtual venue for men who have sex with men to seek sexual partners, with a high potential threat to spread sexually transmitted infections (STIs). However, the online sex-seeking use and its risk causing STIs spread remain unclear among heterosexual men. We conducted a cross-sectional study to investigate the use of online sex-seeking venues and the related risky sex activities (e.g. condomless sex, quick sex) in STIs clinics in Guangdong, China.

**Methods:**

These STIs clinics were involved in the Guangdong governmental sentinel network and we recruited heterosexual men (age >  = 18) between March and August 2018. Multivariable logistic regression models were used to identify the factors associated with online sex-seeking use and risky sex activities with online sex partners.

**Results:**

191 of 2,154 participants (8.9%) ever sought sex online. Among users,16.8% met their partners in-person within 24 h, 31.4% engaged in condomless sex with their last online partner. Online sex-seeking was positively associated with a) Ever been diagnosed with STIs (a*OR* = 3.0, 95%CI:2.0–4.6), and b) Had casual sex in the last three months (a*OR* = 3.3, 95%CI 2.4–4.6). Condomless sex with the last online partner was negatively associated with the correct answer of “Having only one partner can reduce the risk to infect HIV” (a*OR* = 0.3, 95%*CI*:0.1–0.8).

**Conclusion:**

In China, online sex-seeking and its related risky sexual activities are not rare among heterosexual men. Future prevention strategies to reduce STIs incidence should especially target heterosexual men engaging in online sex-seeking.

**Supplementary Information:**

The online version contains supplementary material available at 10.1186/s13690-022-00903-5.

## Background

The use of the internet has dramatically changed people’s social behaviors in the twenty-first century. Specifically, the advantage of efficient communications through the internet has reinforced the behaviors of seeking casual partners through dating websites and apps as well as other social media [1–6]. While the Internet extended people’s social contact, previous studies indicated that the uses of such platforms may facilitate the spread of sexually transmitted infections (STIs) [1, 6–10]. This risk of STIs transmission in online sex-seeking may be explained by the greater number of sexual partners [7, 11] and a higher likelihood of practicing unprotected intercourse among online sex seekers than non-online sex-seeking users [7, 12]. For example, evidence from USA showed the early syphilis outbreak during 2007–2013 was associated with meeting partner online and the number of sex partner acted as an intermediate variable between online resources use and early syphilis infection [11]. Therefore, understanding the profiles of online sex-seeking use and its related sexual activities is important for the development of prevention measures for HIV/STIs.

Although a large number of existing research have reported the use of online sex-seeking and characteristics of its sexual activities among men who have sex with men (MSMs) [2, 7, 13, 14], few studies focused on heterosexual men [15]. More attention should be given to the risk associated with sexual activities of heterosexual men because these males can eventually transmit the HIV/STIs from casual partners to their permanent partners. More importantly, the STIs including Chlamydia trachomatis (CT), Neisseria gonorrhea (NG), Syphilis, and Trichomoniasis have continuously and severely affected human’s morbidity as well as heterosexual men’s quality of life worldwide. In extreme cases, the STIs would result in female infertility, adverse birth outcomes (e.g. spontaneous abortion and stillbirth), and congenital infection [16]. However, the existing evidence still doesn’t indicate a clear association between online partner seeking and condom use or STI status among seekers practicing heterosexual sex [15]. Given the neglected risk of heterosexual men’s online sex-seeking behavior, we urgently need to comprehensively investigate the online sex-seeking behavior pattern and its related sexual behaviors that influence the acquisition and transmission of HIV/STIs infection for re-designing prevention strategies.

We hereby conducted a cross-sectional study to explore the risk of HIV/STIs acquisition and transmission associated with online sex-seeking among heterosexual men in Guangdong, China. The specific objectives of this study include: 1) investigating the profiles of online sex-seeking use among heterosexual men; 2) describing the characteristics of specific sexual behavior among online sex-seeking users; and 3) exploring the factors associated with online sex-seeking, condomless sex, and quick sex.

Concerning China has the largest number of Internet users (approximately 0.83 billion users) among the world population in 2018, the site selection in this study is critical [17]. Guangdong ranked 1^st^ in the Internet development index among 31 Chinese cities. Meanwhile, Guangdong has the greatest amount of new infections in syphilis and NG in China in 2018, with 56,180 and 31,262 new reported cases [18]. A large number of Internet users and STIs cases in Guangdong can provides a unique opportunity to clarify the profiles of online- sex seeking use and its threat to the STIs epidemic.

## Methods

### Study design and setting

We conducted this cross-sectional survey in Guangdong, China from March to August in 2018, which was a sub-study of the Guangdong governmental sentinel surveillance programme on STIs. The Guangdong governmental sentinel surveillance network was established in 2015 and comprised 10 cities (Zhuhai, Dongguan, Foshan, Jiangmen, Qingyuan, Shaoguan, Jieyang, Shantou, Maoming, Zhanjiang) with a high burden of STIs (see supplementary table S 1). The 10 cities were located in the Pearl River Delta, East, West, and North of Guangdong, which can reflect economic and geographic diversity in Guangdong. Their locations are shown in supplementary Figure S 1. In the surveillance programme, 1–3 STIs clinics at the prefecture-level city were selected according to the number of their outpatients. A minimum sample size of 200 to 250 was required for each site [19].

In this survey, participants were specifically asked whether they ever found sexual partners online. If participants reported any use of online venues to find sexual partners, they were classified as online sex-seeking users, while others were categorized as non-users. The online venues contained Weibo, Website, Blog, and Apps. Online partners only referred to female partners that our participants met online.

### Participants eligibility

#### Inclusion criteria

According to the sentinel surveillance protocol [19], the eligible subjects need to be men at least 18 years old who had sex lives and provided informed consent in favor of participation.

#### Exclusion criteria

In the sentinel surveillance programme, male visitors who visited this clinic for other diseases (e.g. reproductive health issues, and dermatology diseases) have been excluded by professional staff because those outpatients were with a lower risk of STIs. Besides, men who already participated into this sentinel surveillance programme in other sites in the same city were also excluded.

**Fig. 1:**
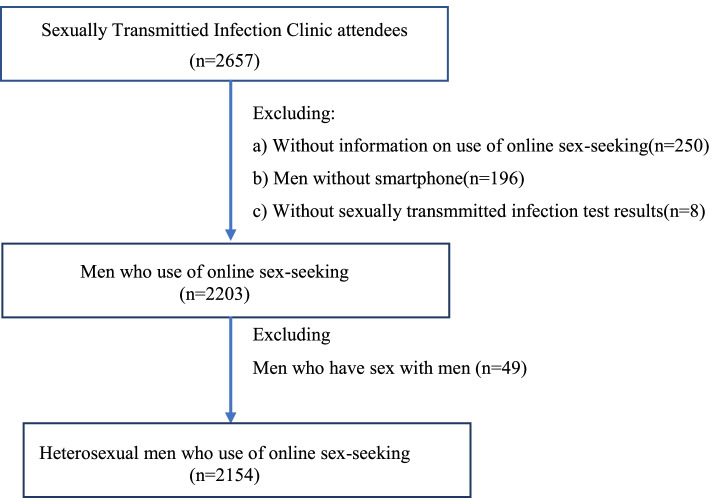
Flowchart of study populations

In our study, we further excluded: a)subjects without information on using online sex-seeking, using a smartphone, and STIs test results, and b) men who ever had sex with men in the past years. The flowchart of the study population was shown as following (Fig. 1).

#### Survey

The sentinel surveillance programme annually observes the prevalence of HIV/STIs (HIV, syphilis, CT, and NG) among STIs clinic visitors. Information from the surveillance includes socio-demographic, sexual behaviors patterns, HIV-related knowledge, and physical conditions. Socio-demographic characteristics include age, residence, marital status, and ethnicity. To understand sexual behavioral patterns, information was collected regarding commercial and casual sex with women in the last three months. The HIV-related knowledge section included eight items, which were shown in supplementary Table S3). Physical conditions were measured by two methods: a) self-reporting the history of HIV/STIs; and b) testing urine and blood from this survey. Urine was self-collected by each eligible participant for CT and NG testing, and blood was collected by professional physicians at the clinics to test HIV and syphilis. More information on testing kits and lab testing methods can be found in Supplementary methods (Table S4).

The information that referred to online sex-seeking use and its related sex activities were as follows: time duration since started to seek sex online, online sex-seeking and the responding number of partners in the last 4 weeks, and the sexual behaviors patterns with the last partner. Sexual behavior patterns included condom use, negotiation about condom use before meeting with the online partners, and ask for HIV status before meeting.

Local STIs clinic staff who assisted the survey were given intensive training. Interview settings had at least 1 private interview/counseling room and a waiting room. After providing verbal informed consent, those who met the eligibility criteria and agreed to participate in the study were referred to a separate, quiet room to complete a questionnaire.

#### Data analysis

Data analyses were conducted in RX64 3.4.2 (R Foundation for Statistical Computing, Vienna, Austria). We used the Chi-square test to compare the difference between categorical factors. Fisher’s exact test was used as an alternative to the Chi-square test when one or more of the cell counts in a cross-table is less than 5.

Univariate and multivariable logistic regression models were used to explore the factors associated with online sex-seeking use adjusting for age, and marital status. The potential factors associated with online sex-seeking and risky sex were included models as categorical variables. For the score of HIV-related knowledge (8 was the total score), we classified it into two categories (< 6, 6–8) and then included them in models. We also separately observed the association between online sex-seeking and condom use and the number of sexual partners.

Subsequently, we performed a sub-analysis to identify the characteristic of users with condomless sex and quick sex. Condomless sex was defined as the sexual behavior that online sex-seekers did not use a condom during the last sexual intercourse with their online partners. Logistic regression models were used to explore the factors associated with condomless sex. Each potential factor was included into model as a categorical variable and further adjusted by age and marital status. Quick sex meant that users met their online partners in person and engaged in sex within one week from the initial meeting. The Chi-square test was used to explore the factor related to quick sex.

## Results

### Social demographic, behavior, and STIs

We recruited a total of 2154 heterosexual men in this study, including 48.0% of which aged over 40 years. Approximately 36.6% and 22.1% of participants self-reported commercial sex and casual sex in the last three months, respectively. More than 74% of heterosexual men agreed that condom use can reduce the risk of HIV infection in sex. Additionally, nearly 9.2% of participants self-reported that they ever suffered STIs. Our lab test showed that the prevalence of HIV, syphilis, CT, and NG were 0.8%, 4.2%, 5.6%, and 4.4%, respectively.

### Use of online sex-seeking

The prevalence of online sex-seeking was 8.8% (191/2154) self-reported by participants (Table 1). Compared with non-online sex-seeking users, the users were generally 1) younger, 2) with a higher prevalence of casual sex, and 3) with a larger proportion of self-reported medical history related to STIs and HIV infection. Besides, our lab test showed that users seem had a higher prevalence of HIV (1.1%, ***P-*** value = 0.26), syphilis (6.8%, ***P-***value = 0.08), and CT ( 7.3%, ***P-***value = 0.33).

**Table 1:** Demographics, behaviors, and physical condition of participants from STIs surveillance sites in Guangzhou, China, 2018 (*N* = 2154) ^a^

Characteristics	Total (*N* = 2154)		Online sex-seeking users (*N* = 191)		Non-online sex-seeking users (*N* = 1963)		*P*-value
	**n**	**%**	**n**	**%**	**n**	**%**	
**Demographic characteristics**							
**Age (years)**							
< 20	56	2.6	6	3.14	50	2.55	** < 0.001**
20–29	569	26.42	69	36.13	500	25.47	
30–39	496	23.03	75	39.27	421	21.45	
40 and above	1033	47.96	41	21.47	992	50.53	
**Residence**							
Guangdong	1892	87.84	173	90.58	1719	87.57	0.27
Other provinces	262	12.16	18	9.42	244	12.43	
**Marital Status**							
Never married	501	23.26	63	32.98	438	22.31	**0.01**
Married or Cohabitation	1600	74.28	124	64.92	1476	75.19	
Divorced or Widowed	53	2.46	4	2.09	49	2.5	
**Ethnicity**							
Han	2134	99.07	189	98.95	1945	99.08	1.00
Minority	16	0.74	1	0.52	15	0.76	
**High-risk behaviors**							
**Engaged in commercial sex in the last 3 months**							
Yes	789	36.63	69	36.13	720	36.68	0.94
No	1365	63.37	122	63.87	1243	63.32	
**The number of commercial partners in the last 3 months**							
1	442	56.02	29	42.03	406	56.39	0.03
> 1	285	36.12	33	47.83	248	34.44	
Missing data	73	9.25	7	10.14	66	9.17	
**Engaged in casual sex in the last 3 months**							
Yes	478	22.19	89	46.6	389	19.82	** < 0.001**
No	1676	77.81	102	53.4	1574	80.18	
**HIV-related Knowledge**							
**Score of HIV-related Knowledge**							
Score (Mean ± SD)	6.24 ± 2.30		7.35 ± 1.50		6.13 ± 2.33		
< 6	660	30.64	19	9.95	641	32.65	** < 0.001**
6–8	1494	69.36	172	90.05	1322	67.35	
**Using condoms correctly can reduce the risk to infect HIV**							
Yes	1709	79.34	183	95.81	1526	77.74	** < 0.001**
No	81	3.76	2	1.05	79	4.02	
Unknown	364	16.9	6	3.14	358	18.24	
**Have sex with only one partner can reduce the risk to infect HIV**							
Yes	1595	74.05	168	87.96	1427	72.69	** < 0.001**
No	121	5.62	3	1.57	118	6.01	
Unknown	438	20.33	20	10.47	418	21.29	
**Physical condition**							
**Self-reported history of STIs**							
Yes	199	9.24	39	20.42	160	8.15	** < 0.001**
No	1955	90.76	152	79.58	1803	91.85	
**Testing result in this survey**							
**HIV**							
Yes	11	0.51	2	1.05	9	0.46	0.26
No	2137	99.21	189	98.95	1948	99.24	
Missing data	6	0.28	0	0	6	0.31	
**Syphilis**							
Yes	89	4.13	13	6.81	76	3.87	0.08
No	2059	95.59	178	93.19	1881	95.82	
Missing data	6	0.28	0	0	6	0.31	
**CT**							
Yes	119	5.52	14	7.33	105	5.35	0.33
No	2008	93.22	175	91.62	1833	93.38	
Missing data	27	1.25	2	1.05	25	1.27	
**NG**							
Yes	90	4.18	6	3.14	84	4.28	0.57
No	2037	94.57	183	95.81	1854	94.45	
Missing data	27	1.25	2	1.05	25	1.27	

^a^*STIs* Sexually Transmitted Infections, *HIV* Human Immunodeficiency Virus, *OR* Odds Ratios, *CI* Confidence Interval, *CT* Chlamydia Trachomatis**,** *NG* Neisseria Gonorrhea

### Sexual activities related to online sex-seeking

Among the men who ever sought sex partners online, 63.0% of them had more than one-year’ experience in online sex-seeking (Table 2). During the study, 55.5% of users found partners online in the last 4 weeks and near 67% of them had one more sexual partners in the last 4 weeks. Moreover, 40.7% of users met their last sexual partner in person within one week, and 31.4% had condomless sex. Besides, before the in-person meeting, 19.9% of them negotiated about condom use, while 89.5% did not inquire about their partner’s HIV status.

**Table 2:** Information and behaviors of online sex-seeking heterosexual men in Guangdong, China (*N* = 191)

**Variables**	n	%
**Time duration since started to seek sex online**		
< 6 months	21	10.99
6 months-1 year	41	21.47
1–3 years	70	36.65
> 3 years	49	25.65
Missing data	10	5.24
**Engaged in finding sexual partners online in the last four weeks**		
Yes	106	55.5
No	81	42.41
Missing data	4	2.09
**The number of sexual partners found online in the last four weeks**		
1	35	33.02
2 to 3	68	64.15
Above 4	3	2.83
**Time-gap of finding the last partners online**		
≤ 1 week	84	43.98
2–4 weeks	51	26.7
> 4 weeks	52	27.23
Missing data	4	2.09
**Time duration between meeting the last sex partner through online and meeting in person**		
≤ 1 day	32	16.75
2–7 days	46	24.08
1–2 weeks	58	30.37
> 2 weeks	52	27.23
Missing data	3	1.57
**Used condoms during last sex with the last partner**		
Yes	128	67.02
No	60	31.41
Missing data	3	1.57
**Negotiation about condom use with the last partner before meeting in person**		
Yes	38	19.9
No	150	**71.49**
Missing data	3	1.32
**Asked for the HIV status of the last partner before meeting in person**		
Yes	17	8.9
No	171	89.53
Missing data	3	1.57

^a^*HIV* Human Immunodeficiency Virus

### Factors associated with online sex-seeking use

Our multivariable regression results (Table 3) showed that online sex-seeking use was positively associated with a) ever been diagnosed with STIs (adjusted Odds Ratio (a*OR* = 3.0, 95%CI: 2.0–4.6), and b) having casual partners in the last three months (a*OR* = 3.3, 95%CI: 2.4–4.6).

**Table 3:** Factors correlated with online sex-seeking use for partner-seeking among heterosexual men in Guangdong, China, 2018 (*N* = 191) ^a^

Variables	Univariate analysis		Multiple analysis^b^	
	**Crude OR**	**95%CI**	**Adjusted OR**	**95%CI**
**Had commercial sex in the last three months**				
No	Ref		Ref	
Yes	0.98	(0.72, 1.33)	1.02	(0.74, 1.41)
**Had casual sex in the last three months**				
No	Ref		Ref	
Yes	3.53***	(2.60, 4.79)	3.32***	(2.40, 4.60)
**Ever been diagnosed with STIs**				
No	Ref		Ref	
Yes	2.89***	(1.96, 4.25)	3.00***	(1.97, 4.56)
**Score of HIV-related knowledge**				
6–8	Ref		Ref	
< 6	1.40	(0.49,378)	1.57	(0.52,4.56)
**Using condoms correctly can reduce the risk to infect HIV**				
No or Unknown	Ref		Ref	
Yes	6.55***	(3.21, 13.41)	5.21***	(2.52, 10.75)
**Having sex with only one partner can reduce the risk to infect HIV**				
No or Unknown	Ref		Ref	
Yes	2.74***	(1.75, 4.23)	2.28*	(1.43,3.65)

^a^*STIs* Sexually Transmitted Infections, *HIV* Human Immunodeficiency Virus, *OR* Odds Ratios, *CI* Confidence Interval

^b^Adjusted for age, marital status, visiting clinic type, and the number of children

^*^P value < 0.05, **P value < 0.01, ***P value < 0.001

### Factors associated with the condomless sex with online partners

As presented in Table 4, having condomless sex with the most recent online partner in the last sex was negatively associated with the correct answers on the HIV-related knowledge: having only one partner can reduce the risk to infect HIV (a*OR* = 0.3, 95%CI: 0.1–0.8). The relationships between condomless sex and characteristics of online sex-seeking users were not significant, such as negotiation about condom use with the last online partner before meeting in person (a*OR* = 1.1, 95%*CI*: 0.5- 2.6).

**Table 4:** Factors correlated with condomless sex with the last partner among online sex-seeking users in Guangdong, China, 2018 (*N* = 77) ^a^

Variable	Univariate analysis		Multiple analysis	
	**Crude OR**	**95%CI**	**Adjust OR**	**95%CI**
**Characteristics of online sex-seeking users**				
Time duration since started to seek sex online				
< 6 months	Ref		Ref	
6 months-1 year	2.61	(0.92, 7.38)	2.39	(0.81, 7.01)
1–3 years	0.70	(0.26, 1.91)	0.61	(0.22, 1.73)
> 3 years	1.05	(0.38, 2.90)	1.04	(0.35, 3.05)
Time-gap of finding the last partners online				
≤ 1 week	Ref		Ref	
2–4 weeks	1.40	(0.68, 2.87)	1.33	(0.63, 2.81)
> 4 weeks	0.54	(0.24, 1.20)	0.51	(0.22, 1.18)
Time duration between meeting the last sex partner through online and meeting in person				
≤ 1 day	Ref		Ref	
2–7 days	1.02	(0.39, 2.67)	1.05	(0.39, 2.86)
1–2 weeks	1.86	(0.76, 4.57)	1.72	(0.68, 4.31)
> 2 weeks	1.25	(0.49, 3.19)	1.28	(0.47, 3.49)
Negotiation about condom use with the last partner before meeting in person				
No	Ref		Ref	
Yes	1.14	(0.54,2.42)	1.14	(0.50, 2.59)
Asked for the HIV status of the last partner before meeting in person				
Yes	Ref		Ref	
No	1.12	(0.48, 2.59)	1.04	(0.42, 2.56)
**HIV related knowledge**				
**Score of HIV-related knowledge**				
6–8	Ref		Ref	
< 6	1.41	(0.49, 3.77)	1.58	(0.53, 4.53)
Using condoms correctly can reduce the risk to infect HIV				
No or Unknown	Ref		Ref	
Yes	0.57	(0.13, 2.83)	0.55	(0.11. 2.75)
Having sex with only one partner can reduce the risk to infect HIV				
No or Unknown	Ref		Ref	
Yes	0.34*	**(0.14, 0.84)**	0.30*	**(0.11, 0.81)**

^a^ *STIs* Ssexually Transmitted Infections, *HIV* Human Immunodeficiency Virus, *OR* Odds Ratios, *CI* Confidence Interval

^b^ Adjusted for age, marital status, visiting clinic type, and having children

^*^P value < 0.05, **P value < 0.01, ***P value < 0.001

### Sexual behaviors patterns of quick sex

Table 5 showed that quick sex was more likely occurred among users who a) had more than 6 months’ experience in using online sex-seeking tools, and b) had no online partners in the last 4 weeks. Compared with users who had sex after a one-week online connection with their partners, the quick sex subgroup has a larger proportion (23.08%, ***P***-value < 0.001) of new online sex-seekers.

**Table 5:** Sex behavior patterns among heterosexual men who met the last partner in person within and over one week after meeting online, 2018

	**Duration between meeting online and meeting in-person**				***P* value**
	**One week or less (*N* = 78)**		**More than one week (*N* = 110)**		
	**n**	**%**	**n**	**%**	
Time duration since to the start of seeking sex online					
< 6 months	18	23.08	9	8.18	** < 0.001**
6 months-1 year	23	29.49	17	15.45	
1–3 years	26	33.33	44	40.00	
> 3 years	11	14.10	38	34.55	
The number of sex partners found online in the last 4 weeks					
0	22	30.21	59	53.64	** < 0.001**
1	18	23.08	17	15.45	
2 to 3	36	46.15	32	29.09	
Above 4	2	2.56	2	1.82	
Used condoms during last sex with the last partner					
Yes	57	73.08	71	64.55	0.28
No	21	26.92	39	35.45	
Negotiation about condom use with the last partner before meeting in person					
Yes	15	19.23	23	20.91	0.92
No	63	80.77	87	79.09	
Asked for the HIV status of the last partner before meeting in person?					
Yes	7	8.97	10	9.09	0.98
No	71	91.03	100	90.91	

## Discussion

Seeking sex through the internet platform has remarkably increased among men who have sex with men in China in recent years, while studies on the use of online sex-seeking venues among heterosexual men remain few. Our study provided compelling evidence that the internet platform has become an important tool to seek sex among heterosexual men, especially for those high-risk populations (e.g. men who had ever been diagnosed with STIs and engaged in casual sex in the last three months). The use of online sex-seeking maybe facilitates HIV/STIs transmission due to the higher rate of condomless sex (31.4%) and quick sex (40.1%) with online sex partners.

To our knowledge, this survey is the first study focusing on online sex-seeking behaviors among heterosexual men in China. Compared with 45% of MSMs seeking sex-partners online and the 59% of them using gay-apps in previous national-wide surveys in China [1, 10], our data showed a lower prevalence (10%) of online sex-seeking among heterosexual men. This rate was also lower than the 14% of patients attending genitourinary medicine clinic in 2002 in United Kingdom [20]. Although online sex-seeking was not so popular in heterosexual men as in MSMs, our findings suggested that the risk of spreading HIV/STIs in Chinese heterosexual men should be highlighted due to the higher rates (31.4%) of condomless sex (Chinese MSMs: 25.4%) and the far lower rates of inquiring condom use (19.9%) (Chinese MSMs: 32.3%) and asking for HIV status (8.9%) (Chinese MSMs: 33.3%) [1]. Our lab results also obtained a higher positivity rate of STIs among users, which implied the necessity of paying attention to heterosexual men in future interventions on seeking sex online.

Additionally, our findings on the difference in characteristics between online sex-seeking users and non-online sex-seeking users implied that this sex-seeking tool was maybe more attractive for those risky populations. Compared to non-users of the online sex-seeking tool, the users were more likely to be young, never get married, and ever been diagnosed with STIs. These features were consistent with findings among MSMs [1] and patients attending genitourinary medicine clinic [20]. The larger proportion of the high-risk population seeking sex online might be explained that young men generally learn how to use new social tools faster and this online sex-seeking tool provides these men an easier way to hide their STIs positive status. We also found that our surveyed users had used online sex-seeking tools for more than 1 year (63.5%) and still had contacted their online partners in the last 4 weeks, which suggested that these users persistently used this sex-seeking tool.

The emerging evidence from behavioral health and chronic disease management show that the Internet is an effective platform to deliver health promotion activities [21]. In this survey, our results found men with a higher score of HIV-related knowledge were more likely to seek sex online (a*OR* = 2.2, 95%*CI*:1.2–4.4), but men knowing the risk of getting STIs through multiple partnerships was negatively associated with having condomless sex (a*OR* = 0.3, 95%CI:0.1–0.6). It suggests that knowledge is a key driver for having protected sex among online sex-seeking users, therefore, strengthening health education should be addressed in the intervention strategies. On the other hand, we did not observe an association between condomless sex and participants knowing the benefit of using condoms to prevent STIs infection. It implies that the awareness of risks may play a more important role in avoiding risky sex behaviors and this awareness should be enhanced in designing future health education materials for online sex-seeking users.

### Strengths and limitations

To our knowledge, our study is one of the very few studies focused on online sex-seeking and its threat to STIs transmission among heterosexual men in China. However, there were several limitations as follows. First, the data was obtained from an organization whose primary mission is to monitor sexually transmitted infections, and there may be a substantial selection bias towards heterosexual men with risky sexual experiences. Because those experiences may have prompted the need and action for STIs’ screening services. Second, the use of online sex-seeking and the related risky behaviors were self-reported, which could be influenced by recall bias, and underreport due to stigma; this might have led to an underestimation of prevalence of online sex-seeking and risk behaviors. Third, despite we observed a higher positivity rate of STIs among the online sex-seeking users, we still can’t identify the impact of online sex-seeking on the spread of STIs through this cross-sectional survey. More longitudinal studies are needed to clarify the drivers on online sex-seeking and the threat of online sex-seeking for STIs spread in the future.

### Implications for policy and practice

Despite the limitations and the low prevalence of online sex-seeking among heterosexual men, we found risky patterns for STIs spread among online sex-seeking users and the relation between users’ HIV-related knowledge and their motivation to avoid risky sex. With the popularity of smart phones and online sex-seeking, its sequential high-risk sexual behaviors in men’s heterosexual sex present interesting challenges and opportunities for developing STIs prevention measures. First, due to the high rate of condomless sex and quick sex among online sex seekers, we suggest to take the online sex-seeking use among heterosexual men and their related sexual behaviors into governmental surveillance programmes in the future. Second, as the right HIV knowledge may decrease the risky sex (e.g. condomless sex), these online platforms should be an important channel to effectively disseminate health messages when seeking partners online. Moreover, our finding is of great importance for men who are not motivated to take a safe sex with online partners. Future studies can be done on identifying the characteristics of high-risk population.

## Conclusion

In China, online sex-seeking and its related risky sexual activities are not rare among heterosexual men. The high proportion of risky sexual behaviors among heterosexual men may facilitate STIs transmission among them. Future prevention strategies to reduce STI incidence should especially target heterosexual men engaging in online sex-seeking.

## Supplementary Information

**Additional file 1:** **Table S1.** The Reporting cases of five surveillance STIs among 10 study settings in Guangdong, China, in 2015 and 2018*. **Figure S1.** The geographic distribution of 10 study settings in this survey. **Table S2.** The number of recruited participants from 10 study settings in Guangdong, China. **Table S3.** The items of HIV-related knowledge in questionnaire. **Table S4.** List of laboratory test method for sexually transmmitied infections. 

## Data Availability

The datasets generated during the current study are not publicly available due to the clinical and confidential nature of the material but are available from the corresponding author on request.

## Abbreviations

HIV: Human immunodeficiency virus
STIs: Sexually transmitted infections
CT: Chlamydia trachomatis
NG: Neisseria gonorrhea
a*OR*: Adjusted Odds Ratio
MSMs: Men who have sex with men
