# Supplementary material for: Online sex-seeking among Chinese heterosexual men who seek care in sexually transmitted infections clinics: a cross-sectional study
Source: Arch Public Health. 2022 Jun 1;80:149. doi: 10.1186/s13690-022-00903-5 (PMC9158293; doi:10.1186/s13690-022-00903-5)
Supplement: Supplementary file 1 — Additional file 1: Table S1. The Reporting cases of five surveillance STIs among 10 study settings in Guangdong, China, in 2015 and 2018*. Figure S1. The geographic distribution of 10 study settings in this survey. Table S2. The number of recruited participants from 10 study settings in Guangdong, China. Table S3. The items of HIV-related knowledge in questionnaire. Table S4. List of laboratory test method for sexually transmmitied infections. [file 13690_2022_903_MOESM1_ESM.docx]

**Supplementary files**

| S1.Table S1. The Reporting cases of five surveillance STIs among 10 study settings in Guangdong, China, in 2015 and 2018 |
| --- |
| S2. Figure S1. The geographic distribution of 10 study settings in this survey |
| S3. Table S2. The number of recruited participants from 10 study settings in Guangdong, China |
| S4. Table S3. The items of HIV-related knowledge in questionnaire |
| S5. Table S4. The labortary test methods for STIs in Guangdong, China |

**Table S1. The Reporting cases of five surveillance STIs among 10 study settings in Guangdong, China, in 2015 and 2018***

| **City** | **Number of Reporting cases in 2015** | **Number of Reporting cases in 2018** |
| --- | --- | --- |
| **Zhuhai** | 4712 | 5793 |
| **Dongguan** | 13489 | 16667 |
| **Foshan** | 15266 | 14818 |
| **Jiangmen** | 5956 | 7098 |
| **Qingyuan** | 5962 | 7367 |
| **Shaoguan** | 3476 | 4103 |
| **Jieyang** | 3031 | 4401 |
| **Shantou** | 3252 | 4076 |
| **Maoming** | 5050 | 5733 |
| **Zhanjiang** | 2631 | 3963 |

***** Five STIs include syphilis, gonorrehoea, chlamydia trachomatics, condyloma acuminatum, herpes.

**
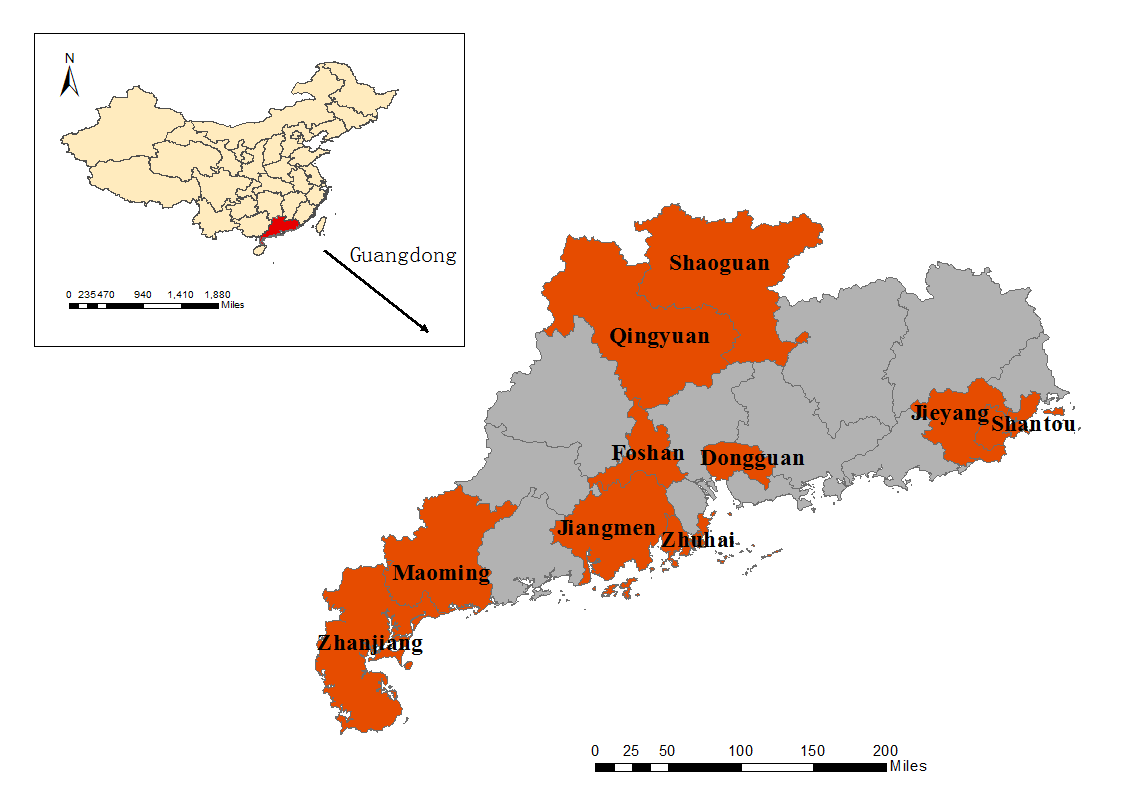
**

**Figure S1. The geographic distribution of 10 study settings in this survey**

| **Table S2. The number of recruited participants from 10 study settings in Guangdong, China** | | |
| --- | --- | --- |
| **City** | **Number of STIs clinic attendees*** | **Number of participants** |
| **Zhuhai** | 250 | 240 |
| **Dongguan** | 249 | 249 |
| **Foshan** | 243 | 236 |
| **Jiangmen** | 250 | 220 |
| **Qingyuan** | 250 | 0 |
| **Shaoguan** | 212 | 212 |
| **Jieyang** | 250 | 248 |
| **Shantou** | 250 | 250 |
| **Maoming** | 250 | 250 |
| **Zhanjiang** | 249 | 249 |

***** STIs means sexually transmitted infections.

| **Table S3. The items of HIV-related knowledge in questionnaire** | | |
| --- | --- | --- |
| **Questions** | **Score for right** | **Score for wrong** |
| 1. A person can look at someone and tell if he or she is living with HIV or has AIDS（Yes/No/Unknown） | 1 | 0 |
| 2. HIV can be easily spread through the bit of a mosquito（Yes/No/Unknown） | 1 | 0 |
| 3. HIV could spread throughsharing dishes with an infected person（Yes/No/Unknown） | 1 | 0 |
| 4. HIV can be transmitted by blood transfusion（Yes/No/Unknown） | 1 | 0 |
| 5. HIV can be transmitted by sharing needle or syringe（Yes/No/Unknown） | 1 | 0 |
| 6. Pregnant women infected with HIV will have babies born with AIDS（Yes/No/Unknown） | 1 | 0 |
| 7. HIV can be prevented by rightly using condoms（Yes/No/Unknown） | 1 | 0 |
| 8 Having sex with only one partner can decrease a person's chance of being infected with HIV | 1 | 0 |
| **Total** | 8 | 0 |

| **Table S4. List of laboratory test method for sexually transmmitied infections** | | |
| --- | --- | --- |
| **Diseases** | **Testing methods** | **Kits** |
| HIV/AIDS | Enzyme-linked Immunoassay (ELISA) test and Western blot assay test | Lizhu Biotech Inc, Zhuhai, China；  HIV Blot 2.2 WB, Wantai Biotech Inc, Beijing, China |
| Syphilis | The rapid plasma regain (RPR) and Treponema pallidum particle agglutination(TPPA) | Lizhu Biotech Inc, Zhuhai, China；  Rongsheng Biotech Inc, Shanghai, China |
| Chlamydia trachomatis | Nucleic Acid Amplification Testing | cobas® 48000 CT/NG Amplification /Detection Kit, Shanghai, China |
| Neisseria gonorrhoeae | Nucleic Acid Amplification Testing | cobas® 48000 CT/NG Amplification /Detection Kit, Shanghai, China |
